# The Characteristics of Patients with Oral Lichen Planus and Malignant Transformation—A Retrospective Study of 271 Patients

**DOI:** 10.3390/ijerph18126525

**Published:** 2021-06-17

**Authors:** Vladimíra Radochová, Romana Koberová Ivančaková, Ondřej Heneberk, Radovan Slezák

**Affiliations:** Department of Dentistry, Faculty of Medicine and University Hospital in Hradec Králové, Charles University, Sokolská 581, 50005 Hradec Králové, Czech Republic; koberovar@lfhk.cuni.cz (R.K.I.); ondrej.heneberk@fnhk.cz (O.H.); slezak@lfhk.cuni.cz (R.S.)

**Keywords:** oral lichen planus, malignant transformation, clinical features

## Abstract

Introduction: Oral lichen planus (OLP) is a chronic inflammatory disease with an unknown etiology rating among oral potentially malignant disorder. The aim of the study was to determine the epidemiological and clinical characteristics of the patients with OLP and rate of malignant transformation. Patients and methods: Data were obtained from the medical records of 271 patients referred to the Oral Medicine Unit at the University Hospital in Hradec Králové diagnosed with oral lichen planus in the period of 2003–2020. The records were retrospectively analyzed. The following clinical data were retrieved from the medical charts: gender, age, systemic diseases, alcohol and tobacco consumption, localization/clinical appearance of lesions, distribution of the lesions, presence of the symptoms, treatment provided and malignant transformation. Results: A total of 271 charts of patients with confirmed diagnosis of OLP were retrospectively analyzed, of whom, 66.4% (180/271) were women and 33.6% (91/271) were men. The mean age of the patients was 56.0 (18.2–85.0) years. The median follow-up of all patients was 15.2 months. Overall, 2 patients (2/271, 0.74%) meeting the above-mentioned criteria for malignant transformation were identified during the follow-up period. Both patients suffered from erosive type OLP and developed squamous cell carcinoma of the tongue. Conclusions: This retrospective study is in concordance with other studies showing the similar profile and clinical features of the patients with OLP. Malignant transformation rate was 0.74%.

## 1. Introduction

Oral lichen planus (OLP) is the chronic or recurrent autoimmune disease of the oral mucosa [1]. Although the etiopathogenesis of OLP is unknown the immune system plays a very important role in disease development [2]. Both antigen-specific and non-specific mechanisms may be involved in the pathogenesis of oral lichen planus [3]. The prevalence of OLP is believed to be around 1% worldwide. Higher prevalence was reported in Europe (1.43%) and lower in India (0.49%) [4]. The prevalence increases significantly and progressively with age, especially over the age of 40 [4]. OLP is characterized by the presence of white lace-like reticular lesions with or without erosive and atrophic lesions [3]. The clinical picture is characterized by changing type/forms of lesions over time. Andreasen was the first who classified OLP and postulated the existence of six different clinical forms [5]. Later, this classification was simplified by other authors who basically divided the clinical forms of OLP into reticular, papular, atrophic and erosive lesions or only for red and white forms [6]. Any intraoral area can be affected, the most commonly affecting the buccal mucosa and lateral tongue; these lesions are almost always present bilaterally. The gingiva and alveolar mucosa are also frequently affected and desquamative gingivitis can be present. The diagnosis of OLP should be based on the combination of clinical and histopathological criteria [7]. Histopathological criteria are: presence of well-defined band such as predominantly lymphocytic infiltrate that is confined to the superficial part of connective tissue, hyperorthokeratosis or hyperparakeratosis, acanthosis, thickening of the spinous layer, liquefaction of the basal layer accompanied by the degeneration of keratocytes and lymphocyte infiltration of the lamina propria [8,9]. It is also critically important to distinguish OLP and other disorders with similar clinical appearance such as oral lichenoid lesions [10]. OLP has been classified as an oral potentially malignant disorder since the first WHO workshop in 2005 [11]. The malignant transformation of OLP was reported to be around 2% [12]. The aim of the study was to evaluate the characteristics of patients with OLP and evaluate malignant transformation cases in the cohort of patients treated and followed up at our institution.

## 2. Materials and Methods

The report was approved by the local ethical committee. All consecutive patients at the Oral Medicine Unit, the Department of Dentistry, Charles University in Prague, Faculty of Medicine and University Hospital in Hradec Králové, Czech Republic, diagnosed with OLP from 2003 to December 2020 were included in the study. All patients had histologically confirmed clinical diagnosis of OLP according to the diagnostic criteria of the World Health Organization (WHO) 1978 modified by van der Meij et al. in 2003 [8]. We excluded patients with oral lichenoid contact lesions caused by an identifiable cause such as a hypersensitivity reaction to dental restorative materials or patients with suspected drug-induced lesions (patients on chronic medication unchanged for one year or longer were eligible). We did not exclude patients with epithelial dysplasia. The following clinical data were obtained from the medical charts: gender, age, alcohol and tobacco consumption, clinical types of lesions and localization of the lesions, presence of the symptoms, extraoral manifestations of lichen planus, presence of systemic diseases, history of the medication, treatment provided (topical corticosteroid in mucosal adhesive paste or intralesional injection or systemic corticosteroid). Malignant transformation case was defined as the development of new histologically confirmed malignant tumor at the site simultaneously affected by OLP. We have also analyzed the patients according to three subgroups: the first group comprises of the reticular and plaque lesions (“white” lesions), the second group comprises of erythematous lesions and desquamative gingivitis (“red“ lesions) and the third group included patients with erosive, ulcerative and bullous lesions (“erosive” lesions). The patient with more types of simultaneous lesions was classified according to the worst clinical presentation (erosive considered as the most serious). We compared the groups for the possible clinical differences. A descriptive statistical analysis was made using Microsoft Excel 2003 (Microsoft, Redmond, WA, USA) and MedCalc 9.5.2.0 (MedCalc Software, Ostend, Belgium). Data were described by absolute and relative frequencies of categorical variables and mean (minimum–maximum) of quantitative variables. For comparison of categorical variables in groups ML, chi-squared test was used, in the case of quantitative variables the Mann–Whitney U test was adopted. *p*-values less than 0.05 were considered statistically significant (all tests two-sided).

## 3. Results

A total of 271 patient’s charts with confirmed diagnosis of OLP were retrospectively analyzed; 66.4% (180/271) were women and 33.6% (91/271) were men, giving a female to male ratio of 1.98:1. All affected patients were white Caucasian. The mean age of the patients at the time of examination was 56.0 (18.2–85.0) years, mean age was 56.1 (18.2–85.0) for women and 50.6 (18.9–74.1) for men and the difference was statistically significantly different (*p* = 0.0009). Detailed data regarding the characteristics of the patients can be found in Table 1.

### 3.1. Systemic Diseases, Medication, Smoking

The most prevalent concomitant systemic disorders included arterial hypertension in 47.2% (128/271), thyroid gland disorders in 19.9% (54/271), diabetes mellitus in 14.0% (38/271), psychiatric diseases 8.1% (22/271) and other various diseases in 42.4% (115/271) patients. Positive allergy history was present in 19.2% (52/271) patients. 70.1% (190/271) patients were on regular medication. 19.2% (52/271) of patients were smokers.

### 3.2. Extraoral Manifestations

Skin lesions were observed in 17.3% (47/271 patients) of the patients (12 men, 35 women). Genital lesions with LP were found in 5.2% (14/271) of patients (4 men, 10 women).

### 3.3. Clinical Types of Oral Lichen Planus and Distribution

The most common type was the reticular form of the OLP which was observed in 259/271 (95.6%) patients. 12/271 (4.4%) patients did not show any reticula and were presented only by other clinical forms. The erosive form was the second most common type with the prevalence of 136/271 (50.2%) patients. Prevalence of the erythematous lesions was 120/271 (44.3%) and plaque form was in 111/271 (41.0%) patients. Desquamative gingivitis was present in 54/271 (19.9%) patients. In all cases of desquamation, there was a combination with another form of OLP. The buccal mucosa being the most common location of each clinical form (248/271, 91.5%), followed by the tongue (93/171, 54.4%), gingiva and alveolar mucosa (44/171, 25.7%), lips (32/171, 18.7%). Lesions of the palate (20/271, 7.4%) and floor of the mouth (12/271, 4.4%) were uncommon. The comparison of subgroups is presented in Table 2.

### 3.4. Treatment

Topical steroids alone were prescribed to 170/271 (62.7%) and in combination with systemic steroids to 19/271 (7.0%) of the patients. 93/271 (34.3%) patients didn´t use any drug (asymptomatic or the symptoms were very small). Dexamethasone gel was used for the local treatment which patients applied several times per day to the most symptomatic areas. In 80/271 (29.5%) cases we used combination dexamethasone gel and depot form of corticosteroid intralesional. Differences in administered treatments are shown in Table 2.

### 3.5. Malignant Transformation

The median follow-up of all patients was 15.2 months (IQR 2.2–48.0 months). Overall, 2 patients (2/271, 0.74%) meeting above-mentioned criteria for malignant transformation were identified during the follow-up period. Both patients suffered from erosive OLP lesions and developed squamous cell carcinoma of the tongue. One patient was 74 years old male, and one was 60 years old female. The female patient was a smoker and the male patient had past history of transient smoking more than 30 years before the diagnosis. Details about both cases with oral squamous cell carcinoma (OSCC) are mentioned in Table 3.

## 4. Discussion

The malignant potential of OLP has been a matter of ongoing long-term debate. Although there is a general agreement that the risk of malignant transformation is not high, the patients with OLP should be followed indefinitely. In a recent meta-analysis of 57 studies by Aghbari et al. an interesting observation was shown. The authors found that during the time period the overall proportion of patients transforming into cancer dropped from 5.9% in 1924 to currently reported 0.9% (CI 0.5–1.3) [13]. This effect is probably given by the lack of diagnostic criteria of OLP before the year 2003 when van der Meij et al. modified the widely accepted WHO criteria for diagnosis of OLP [8]. The strict inclusion criteria are of paramount importance. The analysis by Idrees et al. stressed the risk of exaggeration of malignant transformation rate when diagnostic criteria are not strictly met [14]. In fact, OLP lesions tend to undergo malignant transformation less frequently than other oral potentially malignant disorders [15]. Our data showed a 0.74% transformation rate which seems to be in good agreement with the expected transformation rate.

One of the critically important issues is to distinguish between patients suffering from OLP and oral lichenoid lesions (OLL). OLL are those, not meeting the strict WHO criteria for OLP (examples include contact lesions, chronic graft versus host disease or drug-induced lesions). Patients with OLL have generally higher malignant potential than those with OLP. González-Moles et al. in a recent meta-analysis reported 1.88% transformation rate for OLL and 1.14% for OLP [16]. On the other hand, some authors even question the clinical importance of distinguishing drug-induced lesions from OLP [17]. This same study by González-Moles et al. also included analysis of patients reported to have oral epithelial dysplasia (oral lichenoid dysplasia, OLD) which may be one of the key factors affecting the transformation rate. In a report by Sheraston et al., presence of OLD at the background of OLP resulted in higher malignant transformation than its absence. The authors reported a 0.49% rate in OLP patients without OLD and 6.81% in patients with OLD present. The average time to malignancy was rather short, 4.6 years [18]. On the other hand, the development of OLD during the course of the OLP seems to be a rather rare event. Guan et al. reported only 0.6% of patients who developed OLD during the course of the OLP disease [19].

Other risk factors of transformation include erosive type, female gender, and tongue localization. According to a systematic review published by Guiliani et al., 42.4% (39/92) of OSCC cases were localized on the lateral side of the tongue with the majority having erosive lesions present [20]. Both of our patients presented with erosive lesions and had tongue involvement. It remains unknown whether infection with human papillomavirus (HPV) represents a risk factor for malignant transformation remains unknown. Recent meta-analysis showed association of OLP with HPV but so far no implication for cancer development [21]. No association of HPV and dysplastic changes in OLP was identified [22]. Our previous study found a significant proportion of OLP being positive for HPV as well as in healthy controls [23]. A Spanish study by Gomez-Armayones et al. found no association of HPV with OLP [24]. Both of our patients were OLP HPV negative.

An interesting study providing a high level of evidence was published by Ramos-García et al. A systematic review of previously published systematic reviews was conducted. The authors included 7 previously published systematic reviews into the pooled analysis. The results confirmed the transformation rate in OLP to be 0.44–2.28%, in OLL 1.88–3.8% and in OLP with dysplastic changes 6.22% highlighting the role of dysplasia presence [25].

Ruokonen et al. reported an interesting analysis of patients with OSCC with OLP, OLL or without any oral premalignant lesion. Another interesting note regarding this study was the fact, that majority of patients with OLP were diagnosed at early cancer stage (73%). This fact underlines the necessity of careful long-term follow-up [26]. The authors also observed much less alcohol and tobacco consumption in patients with previous diagnosis of OLP or OLL which stresses the etiological role of OLP and OLL in cancer development. Another study conducted by Aguirre-Urizar et al. with 384 OLP and OLL patients and 10 cases of OSCC found 80% of patients affected by OSCC were non-smokers [27].

Insufficient data exist about the difference between survival rates of patients with OSCC arising from OLP. Muñoz et al. reported better, although not statistically significant survival of patients with OLP-related OSCC (119 vs. 42 months, *p* = 0.201) [28]. Bonnardot et al. reported shorter survival for OLP related OSCC (HR 1.43 CI 0.65–3.13) [29]. Analysis of Gonzáles-Moles et al. points to the fact that only these two trials reported the survival of patients with conflicting results. The meta-analysis did not show any meaningful difference in survival of patients with OLP-derived OSCC and OSCC without OLP (HR 1.00, CI 0.52–1.92) [30].

According to a consensus report published by Warnakulasuriya et al. on behalf of WHO Collaborative Center for Oral Cancer, OLP, OLL as well as oral chronic graft versus host disease represent oral potentially malignant disorders that are clearly associated with the risk of malignant transformation and should be followed accordingly [31].

## 5. Conclusions

We conclude that the malignant transformation rate of OLP in middle European population is not high (0.74%). Nevertheless, OLP patients should be closely monitored for potential development of OSCC to ensure early diagnosis.

## Figures and Tables

**Table 1:** Basic characteristic of all patients.

Variable		All (N)	%	Males	%	Females	%	*p*-Value *
Age (mean, range)		56.0	(18.2–85.0)	50.6	(18.9–74.1)	56.1	(18.2–85.0)	0.0009
Female gender		180	66.4	0	0.0	180	100.0	
Male gender		91	33.6	91	100.0	0	0.0	
Systemic disorders								
	Arterial hypertension	128	47.2	34	37.4	94	52.2	0.0289
	Diabetes mellitus	38	14.0	12	13.2	26	14.4	0.9232
	Cardiac disease	29	10.7	6	6.6	23	12.8	0.1779
	Psychiatric disease	22	8.1	0	0.0	22	12.2	0.0012
	Thyroid gland disease	54	19.9	4	4.4	50	27.8	<0.0001
	Other	115	42.4	28	30.8	87	48.3	0.0085
Confounding factors								
	Drugs	190	70.1	50	54.9	140	77.8	0.0002
	Allergies	52	19.2	15	16.5	37	20.6	0.5218
	Smoking	52	19.2	19	20.9	31	17.2	0.5706
	Amalgam restorations	205	75.6	74	81.3	131	72.8	0.1624
	Artificial crowns	105	38.7	34	37.4	71	39.4	0.8413
	Other restorations	36	13.3	8	8.8	28	15.6	0.1738
Subjective symptoms								
	Burning on food ingestion	182	67.2	51	56.0	131	72.8	0.0085
	Constant pain	71	26.2	17	18.7	54	30.0	0.0636
Extraoral lesions								
	Skin	47	17.3	12	13.2	35	19.4	0.2648
	Genital	14	5.2	4	4.4	10	5.6	0.0907
Lesions distribution								
	Buccal	248	91.5	84	92.3	164	91.1	0.9179
	Alveolar ridge	97	35.8	37	40.7	60	33.3	0.2919
	Tongue	152	56.1	50	54.9	102	56.7	0.8886
	Palate	20	7.4	4	4.4	16	8.9	0.2756
	Lips	60	22.1	22	24.2	38	21.1	0.6752
	Mouth floor	12	4.4	5	5.5	7	3.9	0.7686
OLP form								
	Reticular	259	95.6	87	95.6	172	95.6	0.7686
	Erosive	136	50.2	35	38.5	101	56.1	0.0089
	Erythematous	120	44.3	33	36.3	87	48.3	0.0785
	Plaque	111	41.0	37	40.7	74	41.1	0.9527
	Ulcerative	37	13.7	9	9.9	28	15.6	0.2733
	Bullous	2	0.7	0	0.0	2	1.1	0.7965
	Desquamative gingivitis	54	19.9	12	13.2	42	23.3	0.0697
Treatment required								
	No treatment	93	34.3	42	46.2	51	28.3	0.0054
	Any treatment administered	178	65.7	49	53.8	129	71.7	0.0054
	Topical steroid	170	62.7	45	49.5	125	69.4	0.0021
	Intralesional steroid	80	29.5	21	23.1	59	32.8	0.1304
	Systemic steroid	19	7.0	6	6.6	13	7.2	0.9518
Malignant transformation		2	0.7	1	1.1	1	0.6	0.2477

* Chi-squared test.

**Table 2:** Comparison of subgroups of patients.

Variable		White	%	Red	%	Erosive	%	*p*-Value *
		N = 106		N = 25		N = 140		
Age (mean, range)		50.9	(18.2–78.5)	53.4	(24.5–74.1)	57.0	(18.9–85.0)	0.0036
Female gender		59	55.7	17	68.0	104	74.3	
Male gender		47	44.3	8	32.0	36	25.7	
Systemic disorders								
	Arterial hypertension	47	44.3	10	40.0	71	50.7	0.458
	Diabetes mellitus	13	12.3	2	8.0	23	16.4	0.4282
	Cardiac disease	11	10.4	2	8.0	16	11.4	0.8693
	Psychiatric disease	7	6.6	2	8.0	13	9.3	0.7474
	Thyroid gland disease	17	16.0	5	20.0	32	22.9	0.4151
	Other	39	36.8	10	40.0	66	47.1	0.2576
Confounding factors								
	Drugs	71	67.0	17	68.0	102	72.9	0.5908
	Allergies	21	19.8	4	16.0	27	19.3	0.2786
	Smoking	24	22.6	7	28.0	19	13.6	0.0834
	Amalgam restorations	84	79.2	19	76.0	102	72.9	0.5122
	Artificial crowns	37	34.9	12	48.0	56	40.0	0.4375
	Other restorations	10	9.4	3	12.0	23	16.4	0.2723
Subjective symptoms								
	No symptoms/occasional burning	55	51.9	1	4.0	0	0.0	<0.0001
	Burning on food ingestion	47	44.3	21	84.0	114	81.4	<0.0001
	Constant pain	4	3.8	3	12.0	64	45.7	<0.0001
Extraoral lesions								
	Skin	15	14.2	4	16.0	28	20.0	0.4785
	Genital	3	2.8	3	12.0	8	5.7	0.1612
Lesions distribution								
	Buccal	97	91.5	20	80.0	131	93.6	0.0809
	Alveolar ridge	23	21.7	12	48.0	62	44.3	0.0005
	Tongue	45	42.5	15	60.0	92	65.7	0.0012
	Palate	2	1.9	5	20.0	13	9.3	0.0036
	Lips	10	9.4	8	32.0	42	30.0	0.0003
	Mouth floor	1	0.9	1	4.0	10	7.1	0.0642
Treatment required								
	No treatment	25	23.6	7	28.0	62	44.3	0.0025
	Any treatment administered	81	76.4	18	72.0	78	55.7	0.0025
	Topical steroid	75	70.8	17	68.0	78	55.7	0.0459
	Intralesional steroid	41	38.7	6	24.0	33	23.6	0.0299
	Systemic steroid	1	0.9	0	0.0	18	12.9	0.0005
Malignant transformation		0	0.0	0	0.0	2	1.4	0.082

* Chi-squared test.

**Table 3:** Description of the cases with malignant transformation.

	Case #1	Case #2
Gender	Female	Male
Age at diagnosis of OLP (years)	56.5	64.6
Age at diagnosis of malignancy (years)	58.9	73.1
Smoking	yes, 10 pack years	stopped 30+ years ago
OLP type	reticular, erosive, erythematous	reticular, erosive
Affected sites	tongue, buccal mucosa	tongue, buccal mucosa
OLP high-risk HPV status	negative	negative
Time to malignancy (months)	28.8	102.1
Type of malignancy	OSCC	OSCC
Initial malignancy site	tongue	tongue
Extent of initial disease	T3N1M0	T3N0M0
Treatment	hemiglossectomy, lymph node resection, adjuvant chemotherapy (cisplatin), adjuvant radiotherapy (66 Gy IMRT tongue + 59.4 Gy lymph nodes)	hemiglossectomy, lymph node resection, brachytherapy
Follow-up after OSCC diagnosis (months)	5.1	10.2
Outcome	death due to progression	alive

OSCC: oral squamous cell carcinoma, IMRT: intensity-modulated radiation therapy, Gy: gray, HPV human papilloma virus.

## Data Availability

The data presented in this study are available on request from the corresponding author.
